# Detoxification mechanisms involved in ivermectin resistance in the cattle tick, *Rhipicephalus (Boophilus) microplus*

**DOI:** 10.1038/s41598-018-30907-7

**Published:** 2018-08-17

**Authors:** Valeria Lis Le Gall, Guilherme Marcondes Klafke, Tatiana Teixeira Torres

**Affiliations:** 1 Departamento de Genética e Biologia Evolutiva, Universidade de São Paulo, Rua do Matão 277, 05508-090 São Paulo, Brazil; 2 Instituto de Pesquisas Veterinárias Desidério Finamor, Estrada Municipal do Conde 6000, 92990-000 Eldorado do Sul, Brazil

## Abstract

The cattle tick *Rhipicephalus microplus* is one of the most important ectoparasites with great sanitary and economic impact for cattle rearing worldwide. Ivermectin is commonly used to control tick populations, but its use over the last 30 years has led to the development of resistant populations of *R*. *microplus*, and a concomitant loss of efficacy. In this context, we aimed to determine the metabolic mechanisms that contribute to ivermectin resistance in a resistant strain of this species. We performed lethal time bioassays with inhibitors of detoxifying enzymes and xenobiotic transporters (four detoxification pathways) using two strains of ticks: a susceptible strain, Mozo, and a resistant strain, Juarez. We used four inhibitors to test the involvement of different families of proteins responsible for detoxification of ivermectin, namely cytochrome P450, esterases, glutathione-S-transferase, and ATP Binding Cassette Transporters. We calculated the synergistic factor for each inhibitor and strain. To different degrees, all tested inhibitors altered the mortality rates in the strain Juarez, indicating that multiple mechanisms are responsible for the resistant phenotype. Detoxification mechanisms mediated by ABC transporters were observed to be the most important. Esterases, glutathione-S-transferases, and cytochrome-oxidases played less important roles in detoxification.

## Introduction

*Rhipicephalus (Boophilus) microplus* (Acari: Ixodidae) (Canestrini, 1887) is a tick species distributed in tropical and subtropical areas, including the Neotropical region in southern Africa and Asia. In the Americas, it is distributed in areas between Mexico and northern Argentina, and affects cattle reared for meat and milk production^1^. The bite causes irritation and stress to hosts, mainly due to the introduction of toxic agents in the saliva during feeding. Infestations in cattle have direct negative effects on health and production, resulting in weight loss and decreased milk production. In Brazil alone, *R*. *microplus* is responsible for the loss of 3.24 billion dollars per year^2^, making it the most harmful arthropod ectoparasite in the industry.

*R*. *microplus* is also an important vector of the protozoan, *Babesia* spp., and the rickettsia species, *Anaplasma marginale*, which cause babesiosis and anaplasmosis, respectively. These infections result in a loss of productivity and are widespread in tropical and subtropical Latin American countries with high levels of humidity, including Brazil, Northern Argentina, Uruguay, Venezuela, Paraguay, Colombia, Bolivia, and the Caribbean region and Mexico^3^. The lesions caused by tick bites are also used by the New World screw-worm flies (*Cochliomyia hominivorax*, Coquerel, 1858) as a substrate for oviposition^4^.

As such, pest control of *R*. *microplus* is a consistently high priority. In Brazil, this is carried out mainly through the application of chemical products. Ivermectin, which belongs to the family of macrocyclic lactones, is one of the most widely used acaricides for controlling tick populations. However, its continuous use has led to the development of resistant lineages^5–8^.

Resistance in arthropods can be caused by various mechanisms, including (1) amino acid substitutions in receptors or enzymes that alter the molecular targets of the acaricides, (2) development of behavioural modifications that allow the individual to avoid the chemical, (3) metabolic detoxification, (4) detoxification by ATP binding cassette transporters, and (5) reduction of the acaricide penetration into the parasite body^9–11^.

In *R*. *microplus*, several mechanisms can be involved in ivermectin resistance, including the physiological capability to detoxify or tolerate toxicants. The metabolic mechanisms of detoxification are mediated by enzyme families, including cytochrome P450, esterase, and glutathione-S-transferases. These enzymes oxidize, hydrolyse, and conjugate the substrate. Other proteins, like ABC transporters, also contribute to the detoxification processes by transporting biotransformed or native toxicants to the exterior of cells. The detoxification of pesticides via increased activities of cytochrome P450, esterases, GST, and ABC transporters are well known in several groups of arthropods, including *R*. *microplus*^12^. In this work, we investigated the detoxification mechanisms operative in the *R*. *microplus* ivermectin-resistant strain (Juarez), compared to that in a susceptible reference strain (Mozo). We analysed the detoxification of ivermectin in both the strains using lethal time bioassays in the presence of inhibitors that block the enzymatic or transporter activity of the proteins under study, allowing the toxic effects of ivermectin to prevail, and, consequently, increase the mortality rate.

Piperonyl butoxide (PBO), S,S,S-tributyl phosphorotrithioate (DEF), diethyl maleate (DEM) and cyclosporin A (CsA) were respectively used as inhibitors of cytochrome P450, esterases, GST and ABC transporters, owing to their ability to bind the proteins inducing conformational changes that prevent their detoxification activity. These inhibitors are, in most cases, specific. For these reasons, they are effective and commonly used to help determine the involvement of various proteins in pesticide resistance^13–16^.

If the activities of cytochrome P450, esterases, GST, and/or ABC transporters are involved in ivermectin resistance, their inhibitors could synergistically enhance the toxicity of ivermectin by exerting a larger effect than predicted for the sum of their individual effects. The choice of inhibitors used in the present study was based in previous reports for different species of arthropods, including *R*. *microplus*, showing that Cyclosporin A, S,S,S-tributyl phosphorotrithioate, diethyl maleate and piperonyl butoxide are, respectively, specific inhibitors of ABC transporters^17^, esterases^13–16^, GST^16–19^, and cytochrome-oxidases^15^. Together, these synergize the effect of various pesticides, including pyrethoids, fipronil, amitraz, and ivermectin.

We demonstrate that the resistance to ivermectin is best explained by an increased activity of the ABC transporters, followed by an increased activity of esterases, cytochrome P450, and GST.

## Methods

### Tick strains

Two *R*. *microplus* strains were used in the bioassays. The ivermectin-resistant strain, Juarez, was isolated in 2010 in the municipality of Jacareí in São Paulo State, Brazil. This multidrug resistant strain is also resistant to cypermethrin, amitraz, chlorpyriphos and fipronil^19^. Engorged female cattle ticks were collected in a ranch where a failure in the control of tick populations with ivermectin was reported. After isolation, the strain was maintained in cattle treated with injectable ivermectin (Ivomec®, Merial) at the label rate (200 µg/Kg) on the same day of infestation. The Mozo strain, originally from Uruguay, is the susceptible reference strain, recommended by the Food and Agricultural Organization^20^ for use in tests for diagnosing the resistance strains in Latin America. It was maintained without any contact with acaricides or other tick strains.

Both colonies were maintained in cattle at the Animal Experimentation Unit of the Instituto de Pesquisas Veterinarias Desidério Finamor (IPVDF), Eldorado do Sul, Brazil. All experimental protocols were approved, and all methods were performed in accordance with the guidelines and regulations of IPVDF-Animal Ethics Committee under the permit #02/2012.

### Determination of ivermectin resistance: Larval immersion test

Both the strains, Juarez and Mozo, were tested for their susceptibility to ivermectin using the larval immersion test described by Klafke *et al*.^8^, with some modifications. A 1.2 mM stock solution of 22,23-dihydroavermectin B1 (Sigma) was prepared in acetone PA ACS (Merck). This stock solution was diluted (1:100) in a diluent containing 1% acetone and 0.02% Triton X-100 (Sigma) to obtain the top concentration of 12 µM. Serial dilutions were prepared to obtain solutions of lower concentrations of ivermectin: 8, 6, 4, 3, 1.7, 1.2, 1, 0.7, 0.5, and 0.2 µM. For the control group, only the diluent was used.

A pool of approximately 100–150 larvae was added to 500 µL of each ivermectin solution in 1.5 mL microtubes and incubated for 10 minutes. Thereafter, the larvae were transferred using a paintbrush to 8.5 × 8.5 cm paper filters that were folded and closed with bulldog clips to form a packet. The packets were incubated for 24 h at 28 °C under 80% relative humidity. Mortality was determined by counting all living larvae, defined as those capable of locomotion, versus the total number of larvae per packet. This assay was carried out in triplicate.

Mortality results were subjected to probit analysis using Polo-Plus software^21^. For each test, the following parameters were determined: lethal concentrations for 50% and 90% mortality (LC50 and LC90), with confidence intervals of 95% (CI 95%), and the slope of the regression line. The resistance ratios (RR50 and RR90) at CI 95% were obtained using the Polo-Plus software employing the formula described by Robertson *et al*.^22^. Comparisons were determined to be significant when calculated CI 95% did not overlap.

### Determination of the non-lethal concentrations of the inhibitors

Four inhibitors were used to determine the possible metabolic detoxification of ivermectin in both tick strains, including piperonyl butoxide (PBO) for cytochrome P450, S,S,S-tributyl phosphorotrithioate (DEF) for esterases, diethyl maleate (DEM) for GST, and cyclosporin A (CsA) for ABC transporters. All compounds were obtained from Sigma-Aldrich.

At high concentrations, these inhibitors may have toxic effects on ticks and other arthropods. These toxic effects are caused by the inhibitors themselves, independently of their synergistic effect. Hence, we defined the maximum concentration of these agents as the concentration yielding a mortality of no greater than 5%, since above this level, the independent effects of ivermectin vs. those of other toxicants would be difficult to isolate. Lethal concentrations of 5% were calculated using Probit analysis with the Polo-Plus software^21^.

The larval immersion tests were carried out in triplicate with the susceptible reference strain, Mozo, using different concentrations of inhibitors diluted in a solution of 1% acetone and 0.02% Triton X-100. The following concentrations of each inhibitor were tested: (a) PBO: 1.88, 3.75, 7.5, 15 and 30 µM; (b) DEF: 0.3, 3, 30 and 300 µM; (c) DEM: 50, 100, 200, 400, 800, 1600 µM. We used the concentration of CsA (15 μM), which represents the maximum non-lethal concentration, determined in a previous study^11^, at which the mortality of larvae was found to be < 5%.

### Lethal time bioassays in the presence of inhibitors

Five test solutions containing ivermectin (12 µM) and inhibitors, at the concentrations experimentally determined as described in the previous section, were used for lethal time bioassays. For each bioassay, four experimental groups were made for each strain. These included: Ivermectin 12 µM (positive control), Diluent (negative control), inhibitor only, and Ivermectin 12 µM + inhibitor.

About 100–150 larvae were incubated with 500 µL of the solutions in 1.5 mL microtubes for 10 minutes, with mortality determined at six time points, viz., 10, 120, 240, 360, 480, and 1440 minutes after the exposure, as described for the resistance test above.

Mortality data were analysed using the Probit model as described previously^23^. Median lethal times (LT_50_) for each treatment and strain were determined, with CI 95%, using Polo Plus software, and the package ‘ecotox’ in the R statistical environment version 3.4.0 (Probit and Logit for comparison)^24^. The synergism ratios (SR) were calculated using Polo Plus, considering the LT_50_ of each strain exposed only to ivermectin 12 µM and LT_50_ of the same strain exposed to ivermectin 12 µM + inhibitor. Plots were generated using the R package ‘ggplot2'^25^.

## Results

### Determination of ivermectin resistance

A toxicological bioassay, the larval immersion test, was used to determine and compare the median lethal concentrations (LC_50_) and 90% lethal concentrations (LC_90_) of ivermectin for the Mozo and Juarez strains. The resistance ratios (RR) of the Juarez strain was calculated from the LC_50_ and LC_90_ values, confirming the ivermectin resistance status of the Juarez strain (RR_50_ of 6.86 and a RR_90_ of 20.79) (Table 1).

**Table 1:** Lethal concentrations of 50 and 90% and resistance ratios of 14–21 days larvae of Mozo and Juarez strains of *Rhipicephalus microplus*, as determined by the larval immersion test.

Strain	N	Slope ± SE	Chi-square	DF	H	LC_50_ (CI95%) µM	LC_90_ (CI95%) µM	RR_50_ (CI95%)	RR_90_ (CI95%)
Juarez	4539	1.69 ± 0.08	136.6	31	4.41	9.79 (8.24–12.1)	56.25 (37.74–101.31)	6.86 (6.22–7.57)	20.79 (16.52–26.17)
Mozo	3474	4.62 ± 0.21	200.71	31	6.47	1.43 (1.25–1.59)	2.71 (2.36–3.29)	—	—

N = number of individuals; SE = standard error; DF = degrees of freedom; H = heterogeneity on the Chi-square goodness of fit test; LC = lethal concentration in µM; CI = confidence interval, RR = resistance ratio.

### Determination of the non-lethal concentration of the inhibitors

**Figure 1:**
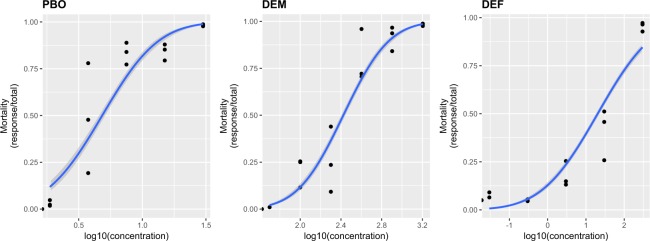
Regression plots obtained for the assays carried out with the larvae of Mozo strain in the presence of the inhibitors piperonyl butoxide (PBO), S,S,S-tributyl phosphorotrithioate (DEF), and diethyl maleate (DEM). This test was carried out to determine the maximum concentration of inhibitors that could be used to test the enzymes. Curves were estimated using a generalised linear model (glm) with a binomial distribution and a “probit” link function. Mortality was modeled as a function of the log-transformed concentration of the inhibitor (in µM). Shaded areas represent 95% confidence intervals.

Prior to the use of the inhibitors in the lethal time bioassays with ivermectin, we determined the maximum non-lethal concentration of three of the four inhibitors that could be used to test the enzymes and the ABC transporters, allowing a maximum mortality of 5%. For CsA, this data was obtained previously^24^. Regression analyses obtained from the bioassays carried out with the larvae of Mozo strain in the presence of the inhibitors PBO, DEF, and DEM (Fig. 1) were used to calculate sublethal doses (LC_5_; CI 95%) for PBO, DEM, and DEF. These were 1.29 (0.58–1.99), 68.92 (43.15–94.67), and 5.36 (0.05–16.99), respectively (Table 2).

**Table 2:** Lethal concentrations obtained from the bioassays carried out with the susceptible Mozo strain of *Rhipicephalus microplus* exposed to the synergists, piperonyl butoxide, S,S,S-tributyl phosphorotrithioate, and diethyl maleate.

Inhibitor	N	Slope ± SE	Chi-square	DF	H	LC_5_ (CI95%) µM	LC_50_ (CI95%) µM
piperonyl butoxide	1079	2.87 ± 0.14	123.41	13	9.49	1.29 (0.58–1.99)	4.84 (3.59–6.19)
S,S,S-tributyl phosphorotrithioate	2165	1.82 ± 0.19	85.33	12	7.11	5.36 (0.05–16.99)	43.16 (10.01–69.16)
diethyl maleate	1536	2.81 ± 0.11	117.21	16	7.33	68.92 (43.15–94.67)	265.24 (213.61–329.29)

N = number of individuals; SE = standard error; DF = degrees of freedom; H = heterogeneity on the Chi-square goodness of fit test; LC = lethal concentration in µM; CI = confidence interval.

### Lethal time bioassays

Lethal time bioassays with inhibitors in combination with ivermectin were carried out to determine the involvement of cytochrome P450, GST, esterases, and ABC transporters in the detoxification of ivermectin. The choice of model (probit or logit) did not affect the results (Supplementary Table 1).

**Figure 2:**
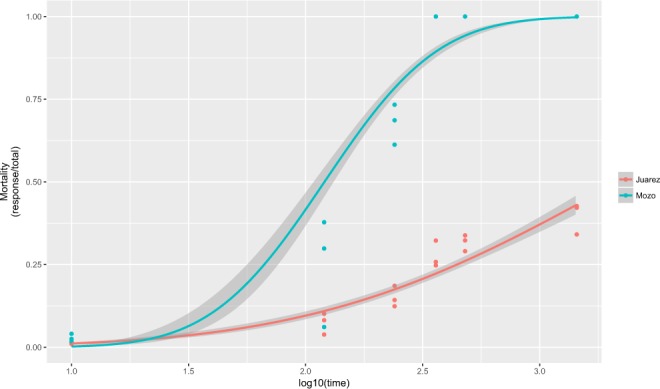
Mortality of the Mozo strain (susceptible) compared to that of the Juarez strain (resistant) exposed to 12 µM of ivermectin at different times. The Mozo strain presented 100% mortality rate at 240 minutes, whereas the Jurez strain presents only 40% mortality in the same period of time. Curves were estimated using a generalised linear model (glm) with a binomial distribution and a “probit” link function. Mortality was modeled as a function of the log-transformed time (in minutes). Shaded areas represent the 95% confidence intervals.

Lethal times for Mozo and Juarez strains exposed to 12 µM of ivermectin were recorded (Fig. 2). A clear difference was observed in the response to treatment with ivermectin across time. After 480 minutes of exposure, all Mozo larvae had died, while the mortality of Juarez larvae had reached only 40%. This was maintained until the end-point at 1440 minutes. The LT_50_ (IC95%) for the larvae of Mozo strain was 120.4 (64.6–173.7) minutes (Table 2). The larvae of the Juarez strain were found to be much more resistant to ivermectin at the 12 µM concentration, with a significantly higher LT_50_ estimated as 2166.7 (1564.7–3393.6) minutes. This observation reinforces the phenotype of ivermectin resistance in the Juarez strain determined through the dose-response experiment (Table 1).

**Figure 3:**
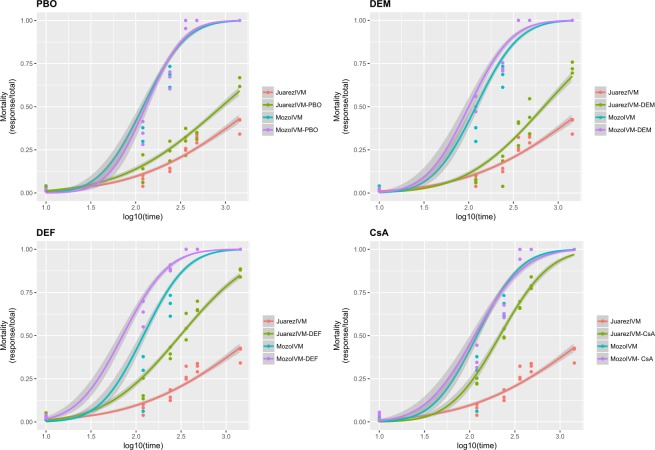
Mortality of Mozo and Juarez strains exposed to ivermectin (IVM) and inhibitors at different times. When Juarez strain was exposed only to IVM, 40% mortality was observed, but under exposure to IVM along with one of the inhibitors, mortality increased. No differences were observed in the mortality of Mozo strain when exposed to IVM alone or in combination with any of the inhibitors, piperonyl butoxide (PBO), diethyl maleate (DEM), S,S,S-tributyl phosphorotrithioate (DEF), or cyclosporine A (CsA). When exposed to IVM along with with CsA, the mortality of Juarez strain reached 100%. This mortality was equal to that of the susceptible strain, Mozo, when exposed to IVM. Curves were estimated using a generalised linear model (glm) with a binomial distribution and a “probit” link function. Mortality was modeled a function of the log-transformed time (in minutes). Shaded areas represent 95% confidence intervals.

The response of the Juarez strain in each of the groups (ivermectin, ivermectin with inhibitors, inhibitors alone, and diluent) in the experiment involving the different inhibitors was compared to that of the Mozo strain (Fig. 3A–D). In each experiment, mortality was determined at six time points, beginning 10 minutes after exposure to the respective solution.

We expected that, in the Juarez strain, at least a part of the resistant phenotype was due to the detoxification mechanisms mediated by enzymes and ABC transporters. If they played a role in the detoxification process, their inhibition would lead to an increased mortality rate, resulting in a diminished LT_50_, and an increased Synergism Ratio (SR). As expected, we observed significant differences in LT_50_ and SR in the resistant strain treated with ivermectin in combination with the inhibitors, or with ivermectin alone (Table 3). The mortality of the control groups (inhibitor only and diluent only) was ≤5% below all the experimental conditions (data not shown).

**Table 3:** Results of the lethal time bioassays carried out with the Mozo (susceptible) and Juarez (resistant) strains of *Rhipicephalus microplus* exposed to ivermectin and synergists.

Strain	Treatment	N	Slope ± SE	Chi- square	DF	H	LT_50_ (minutes) (CI95%)	SR (CI95%)
Juarez	Ivermectin 12 µM	2971	0.98 ± 0.06	36.01	16	2.25	2166.7 (1564.7–3393.55)	—
	+PBO	2662	1.14 ± 0.06	55.05	16	3.44	902.44 (705.75–1231.65)*	2.4 (1.83–3.16)
	+DEM	2927	1.44 ± 0.07	131.52	16	8.22	693.02 (518.7–1030.11)*	3.13 (2.41–4.05)
	+DEF	2514	1.55 ± 0.07	76.81	16	4.8	309.2 (252.16–378.36)*	7.01 (5.45–9.01)
	+CsA	1638	2.29 ± 0.12	67.74	16	4.23	216.02 (175.19–258.71)*	10.03 (7.8–12.89)
Mozo	Ivermectin 12 µM	1636	2.62 ± 0.14	257.37	16	16.09	120.36 (64.61–173.73)	—
	+PBO	1259	2.95 ± 0.19	227.68	16	14.23	129.05 (65.22–183.03)	0.93 (0.81–1.08)
	+DEM	1199	2.64 ± 0.17	63.98	16	3.99	100.13 (71.03–127.86)	1.2 (1.02–1.42)
	+DEF	1502	2.49 ± 0.12	35.927	16	2.25	70.94 (56.99–85.16)	1.69 (1.45–1.99)
	+CsA	1043	2.3 ± 0.14	104.1	16	6.51	113.37 (73.77–154.86)	1.06 (0.9–1.25)

N = number of individuals; SE = standard error; DF = degrees of freedom; H = heterogeneity on the Chi-square goodness of fit test; LT = lethal time in minutes, SR = synergism ratio, CI = confidence interval, *values significantly different from the LT50 calculated for the treatment with ivermectin only, within the tests for the same strain.

Lower levels of detoxification/excretion of ivermectin were expected to be present in the susceptible Mozo strain. However, when this strain was tested, no significant differences were observed in the mortality across time and in the LT_50_ values between the groups treated with ivermectin (1826.8 minutes, 1162,47–4018,62) and ivermectin combined with inhibitors (Fig. 3A–D; Table 3).

In the Juarez strain, the lowest synergism of the four inhibitors tested was observed for PBO (SR = 2.4). In this case, we also observed the higher LT_50_ (CI 95%) value (902.44; 705.75–1231.65) minutes among the combinations of ivermectin and inhibitor. The mortality after 24 hours (1440 minutes) increased more than 20% in the resistant strain exposed to ivermectin in combination with PBO (Fig. 3A). However, in the susceptible strain, no significant differences were observed either in the SR, LT_50_, or mortality rate when exposed to the same treatment (Table 3, Fig. 3A).

An increased toxicity of ivermectin, with an SR of 3.13, was observed in the treatment that included DEM (Table 3). In the treatment with a combination of ivermectin and DEM, the LT_50_ (CI95%) value was significantly lower (693.02; 518.7–1030.11) than in the treatment with ivermectin alone (2166.7; 1564.7–3393.55) (Table 3). The mortality rate of the Juarez larvae at 24 hours was more than 30% higher with the addition of DEM (Fig. 3B). The Mozo strain did not show any difference in the SR, LT_50_, or mortality rate among the treatments (Table 3, Fig. 3B).

The resistant strain treated with DEF and ivermectin showed a significant 7-fold reduction in the LT_50_ (2166.7; 1564.7–3393.6) compared to that observed for the treatment with ivermectin without the inhibitor (309.2; 252.16–378.36) (Table 3), and the mortality rate for ivermectin when combined with DEF was almost 50% higher than with ivermectin alone after 24 hours (Fig. 3C). Under the same conditions, the ivermectin toxicity was slightly increased on the Mozo strain, with a rediction in the LT50 (120.4 ;64.6–173.7) compared to that observed for the treatment without the inhibitor (70.9; 56.9–85.2). Nevertheless, this difference was not significant. The larvae mortality after 24 hours was 100% for both treatments and the SR was 1.69 (Table 3, Fig. 3C).

The highest SR (10.03), and the lowest LT_50_ (216.02 minutes) values in the Juarez strain were observed for ticks treated with a combination of ivermectin and CsA. The median lethal time of ivermectin in the presence of the inhibitor of ABC transporters (216.02; 175.19–258.71) was significantly lower than the LT_50_; IC95% of the same strain exposed to ivermectin alone (2166.7; 1564.7–3393.55) (Table 3). Interestingly, the LT_50_ for the for the combination IVM + CsA approximated the LT_50_ of ivermectin alone in the Mozo strain, providing evidence for an almost complete reversion of the resistant phenotype of the Juarez strain. The synergistic effect of CsA was demonstrated by a 60% increase in mortality of the strain exposed to the ivermectin and CsA combination when compared to the same strain exposed only to ivermectin after 24 hours. Moreover, the diluent and CsA were not responsible for the mortality because the individuals treated with either of these had a mortality rate lower than 5%. Rather, the mortality was caused by ivermectin when the activity of the ABC transporters was inhibited by CsA. Mortality after 24 hours changed from 40% in the case of ivermectin alone to almost 100% when used in combination with CsA (Fig. 3D). On the other hand, the Mozo strain showed no differences in the LT_50_ or mortality rate and the SR was calculated to be 1.06 (Table 2, Fig. 3D).

These results are strong evidence of the involvement of the four detoxifying protein families in the resistant phenotype of the Juarez strain. When these proteins were inhibited, there was an increase in the susceptibility of the resistant strain. The addition of inhibitors did not change the susceptibility of the Mozo strain, indicating that the protein families tested have no or little involvement in the response to the ivermectin exposure in this strain.

## Discussion

Resistance to acaricides is a widespread phenomenon in populations of the cattle tick, *R*. *microplus*. Klafke *et al*.^13^ tested the ivermectin resistance levels of 104 cattle tick samples from different ranches of southern Brazil and found that 60.6% of the samples were resistant to this compound.

The molecular basis of ivermectin resistance in *R*. *microplus* is not completely understood. The enzyme families of cytochrome P450, esterases, GSTs, and ABC transporters involved in detoxification processes are well known for their crucial involvement in the resistance mechanisms in several arthropods, including ticks and other acari. In this context, we wanted to investigate the detoxification mechanisms underlying ivermectin resistance by testing four inhibitors for four different detoxification proteins. In this study, we used two strains of the same species: Mozo, an ivermectin susceptible strain, and Juarez, an ivermectin resistant strain. The resistance of the Juarez strain was 6.86-fold higher than that of the Mozo strain (Table 1).

As evidenced from our results, cytochrome P450, GST, esterases, and ABC transporters are involved in the detoxification process of ivermectin in the R. microplus resistant strain Juarez to different degrees. The most important role is played by the ABC transporters, followed by esterases, GSTs, and cytochrome P450 families of detoxification enzymes.

Metabolic resistance results from the increased breakdown and excretion of insecticide molecules, a mechanism that has probably evolved from an ancestral ability to degrade dietary toxins^23,26^. Hence, these mechanisms are expected to be present at a lower level even in the susceptible strain. The enzymes and the transporter tested here, however, were not involved in the detoxification in the susceptible strain. If there is a lower level of ivermectin detoxification in the Mozo strain, it is probably mediated by different proteins.

Cytochrome P450 is a superfamily of enzymes, also called CYP, present in all organisms. More than 200 CYP gene families have been described. These code for proteins with conserved structural features, even when their sequence similarity is relatively low^27^. Cytochrome P450 enzymes play different roles in eukaryotes, including carbon assimilation, biosynthesis of hormones and cellular components, and xenobiotic degradation via an oxidation reaction that takes place through conformational changes. When the inhibitor PBO binds a cytochrome P450, these changes do not occur and the enzyme is unable to oxidize the pesticides. In our *in vivo* experiments, the inhibition of cytochrome P450 led to an increase in mortality of the resistant strain. A 20% increase in the mortality rate after 24 hours was observed when the resistant strain was exposed to ivermectin and PBO compared to the same strain exposed only to ivermectin (Fig. 3A). Other studies demonstrated that cytochrome P450 is involved in the resistance of ticks to various acaricides.

Metabolic resistance to synthetic pyrethroids due to cytochrome P450 activity has been previously observed in the Coatzacoalcos Mexican population of *R*. *microplus*^19^, and also been thoroughly documented in insects. They mediate resistance to pyrethroids in *Musca domestica*^28^, *Culex quinquefasciatus*^29,30^, *Anopheles funestus*^31,32^ and *Anopheles gambiae*^33^, to name a few. In *Drosophila melanogaster*, a single cytochrome P450 gene, Cyp6g1, is responsible for resistance to DDT^34^. In the Colorado potato beetle, *Leptinotarsa decemlineata*, silencing by RNA interference of one cytochrome P450 gene (Cyp4q3) increased the susceptibility of resistant beetles to imidacloprid, indicating the contribution of P450 to the metabolic resistance^27^. For some species, the involvement of P450 in the pesticide resistance was revealed by the specific inhibition of these enzymes by PBO. In scabies mites (*Sarcoptes scabiei*), lethal time bioassays with permethrin in combination with PBO revealed reduced survival of individuals exposed to the combination when compared to individuals exposed only to permethrin^14^ Detoxification of pyrethroids because of the activity of microsomal monooxygenases was also seen in the spotted spider mite (*Tetranychus urticae*)^35^. In the German cockroach, *Blattella germanica*, PBO was tested, but had a small effect on resistance to carbamate insecticide^36,37^. In *An*. *gambiae*, PBO also significantly enhanced the susceptibility to pyrethroids^38^. It should be noted that results from PBO assays must be interpreted with caution, since PBO is not entirely specific for cytochrome P450. It may also cause a reduction in the cuticular penetration rate of pesticides^30,39,40^. However, if PBO were, in fact, decreasing the penetration of ivermectin in our assays, similar levels of synergism would be observed in the susceptible strain, which was not the case.

GSTs represent a group of multigene isozymes involved in detoxification of endo- and xenobiotics. The cytosolic GSTs are classified into five gene families, namely alpha (α), mu (µ), pi (Π), theta (θ), and sigma (σ), based on their sequence similarity and cross-immunoreactivity^40^. GSTs are present in all aerobic eukaryotes as catalysts of the conjugation of the reduced form of glutathione (GSH) with molecules with an electrophilic centre. A thioether bond is formed between the sulfur of GSH and the substrate, making resulting conjugates more water soluble and facilitating their excretion from cells^40^. Like every enzymatic reaction, its ability to catalyse is made possible by the conformational changes that take place during the reaction. These changes might be affected by the binding of DEM, preventing the conjugation of ivermectin with the reduced GSH. In the ivermectin-resistant strain, Juarez, treatment with the combination of ivermectin and DEM increased the mortality by 30% in comparison to mortality in the same strain treated only with ivermectin after 24 hours. The metabolic resistance mediated by GST against deltamethrin was also demonstrated via synergistic effects of DEM in populations of *R*. *microplus* from New Caledonia^15^. Further, Li *et al*.^16^ found that the resistance to amitraz was mediated by GST in their studies with DEM. In *S*. *scabiei*, an inhibition experiment demonstrated that individuals exposed to a combination of permethrin and DEM had a higher mortality rate compared to individuals exposed only to permethrin^14^. The use of DEM in *Cu*. *quinquefasciatus* strains resistant to pyrethroids also indicated that GST-mediated metabolism contributed to resistance^33^, and in the tarnished plant bug, *Lygus lineolaris*, the use of DEM revealed the involvement of GSTs in the resistance to malathion^41^.

Esterases hydrolyse ester bonds, cleaving xenobiotics and generating acid and alcohol as metabolites. This process involves a nucleophilic attack on the acaricide followed by the liberation of the acid and a return to the active state^42^. In multiple species (e.g. *Lucilia cuprina* and *Co*. *hominivorax*), single amino acid substitutions result in kinetic changes in the esterase E3 gene, which reduce the native ali-esterase activity of the enzyme and enhance OP hydrolase activity^43–45^. Like other metabolic mechanisms, hydrolysis depends on conformational changes that are prevented when an inhibitor such as DEF binds the enzyme. In fact, the addition of DEF in the ivermectin formulation increased the mortality rate by almost 50% at 24 hours in the resistant strain of *R*. *microplus* compared to the mortality of the same strain treated only with ivermectin. In the Coatzacoalcos strain of *R*. *microplus*, the use of triphenyl-phosphate (TPP), an esterase inhibitor, indicated the involvement of esterases in the resistance to synthetic pyrethroids^13^. In *R*. *microplus*, the resistance against deltamethrin and amitraz mediated by esterases was also demonstrated via the synergistic effects of TPP^18,19^. In *S*. *scabiei*, inhibition with DEF in lethal time bioassays with permethrin demonstrated the involvement of esterases in the detoxification process^14^. The detoxification of pyrethroids by esterases was also demonstrated in *Tetranychus urticae* mites^35^. In the German cockroach, *B*. *germanica*, treatment of adult males with the DEF esterase inhibitor increased carbamate toxicity 6.8-fold, implicating esterases as important enzymes in the metabolic resistance to carbamate insecticide^36^. In another study in *B*. *germanica*, the DEF synergist completely eliminated the resistance to carbamates^46^. DEF also increased neonicotinoid toxicity 5.58 times in a resistant strain of the cowpea aphid, *Aphis craccivora*, compared with the susceptible strain^37^.

Finally, ABC protein family is the largest transporter family and is present in all taxonomic kingdoms^47^. These transporters are able to translocate solutes across the cellular membrane, and require the binding and hydrolysis of ATP. The ABC transporters typically have four domains, with two transmembrane domains (TMD) associated with two ATP binding domains (NBD), although there are proteins with a different number of domains (half transporters). Because of their ability to transport multiple substrates, including drugs, many ABC transporters are involved in multidrug resistance^48^. For instance, the P-glycoproteins (P-gps) of the ABCB subfamily, was the first ABC transporter identified as having a role in multidrug resistance in tumour cell lines^48,49^. P-gps have also been implicated in resistance to multiple insecticides and acaricides^50^. The acaricides interact with the TMD domains on the inner face of the membrane, inducing a conformational change that initiates ATP hydrolysis and opens the central pore of the molecule. The substrate passes through the pore to reach the extracellular environment in a mechanism that remains unclear^47^. The binding of CsA to these transporters prevents the translocation of toxicants from the intracellular to the extracellular environment. In the Juarez strain, as revealed here, it allowed the toxic mechanisms of ivermectin to occur, leading to a 60% increase in mortality of the resistant strain and reducing the time necessary to kill 50% of tick larvae 10-fold.

Our lethal time bioassays demonstrated the principal role of ABC transporters in the detoxification of ivermectin in the Juarez strain of *R*. *microplus*. This result is supported by those of another study, which revealed increased toxicity of ivermectin when Juarez strain *R*. *microplus* larvae were pre-exposed to CsA in a larval packet test^17^. Pohl *et al*.^17^ also demonstrated an increase in ivermectin toxicity in engorged adult female ticks of the Juarez strain fed on blood treated with ivermectin and CsA, leading to diminished oviposition and egg viability. The potential involvement of ABC transporters in pesticide resistance was observed in several arthopods, including resistance to carbamates in the green peach aphid, *Myzus persicae*^51^, to abamectin in *D*. *melanogaster*^52^, to ivermectin in *Culex pipiens*^53^, and to neonicotinoids in *Apis mellifera*^54^. CsA was used to uncover the role of ABC transporters in ivermectin resistance in the brown dog tick *Rhipicephalus sanguineus*^46^ and the midge *Chironomius riparius*^55^.

The increased mortality of the Juarez strain is observed not only when esterases and ABC transporters are inhibited, but also when cytochrome P450 and GSTs are inhibited by their specific synergists. Even when the increase in mortality is not as pronounced, it indicates that these enzyme families play a role in the detoxification of ivermectin in *R*. *microplus*. The enzymes and transporters tested are known to interact with a wide variety of pesticides in addition to ivermectin. As such, they could also be used for the detoxification of multiple pesticides. A house fly strain resistant to permethrin, for instance, also developed cross-resistance to different insecticides within and outside the pyrethroid group, with resistance to beta-cypermethrin, cypermethrin, deltamethrin, propoxur, and fipronil all mediated at least in part by P450 detoxification^55^. In the Mediterranean fruit fly *Ceratitis capitata*, resistance to malathion mediated by esterases conferred cross-resistance to other organophosphates, the carbamate carbaryl, the pyrethroid lambda-cyhalothrin, and the benzoylphenylurea derivative lufenuron^56^. ABC transporters have also been described as a generic mechanism that confers resistance to multiple chemicals, including 27 different insecticides/acaricides belonging to nine distinct chemical classes and with several groups with different modes of action, including carbamates, macrocyclic lactones, neonicotinoids, organophosphates, pyrethroids, cyclodienes, benzoylureas, phenylpyrazoles, and DDT^57^. The Juarez strain is also resistant to cypermethrin, amitraz, chlorpyriphos and fipronil^19^, and it remains to be tested if this is due to cross-resistance or if there are additional mechanisms present in this strain.

These results provide evidence of the action of detoxification mechanisms in *R*. *microplus* resistant to ivermectin, contributing to the understanding of the molecular basis underlying the ivermectin-resistant phenotype. This knowledge can aid in the search for new strategies to deal with resistance to ivermectin in the field. For instance, the inhibitors tested, or other molecules with similar effects, could be introduced in commercial formulations of ivermectin. Other chemical derivatives that could escape the action of ABC transporters or esterases could also be new targets for agrochemical development.

The differences in the activities of the proteins studied here could be caused by variations in the structure, sequence, or expression between the susceptible and resistant strains. Investigations on the substitutions in the coding sequences of genes belonging to these four families, as well as on their expression levels, might help to establish the causal relationships between gene function and metabolic resistance to ivermectin.

## Conclusions

We carried out lethal time bioassays to determine the involvement of detoxification mechanisms in ivermectin resistance in the southern cattle tick, *R*. *microplus*. The mortality of larvae of a resistant strain, Juarez, was significantly higher when exposed to ivermectin in combination with inhibitors of four protein families compared to mortality when exposed to ivermectin alone. These results reveal that ABC transporters and proteins of the esterase family play a major role in the detoxification of ivermectin, followed by the GST and cytochrome P450 family of enzymes.

## Electronic supplementary material

Supplementary Dataset 1

## Data Availability

The datasets generated during and/or analysed during the current study are available from the corresponding author on reasonable request.
